# Reaction Time “Mismatch Costs” Change with the Likelihood of Stimulus–Response Compatibility

**DOI:** 10.3758/s13423-022-02161-6

**Published:** 2022-08-25

**Authors:** Megan E. J. Campbell, Chase S. Sherwell, Ross Cunnington, Scott Brown, Michael Breakspear

**Affiliations:** 1 School of Psychological Sciences, University of Newcastle, Callaghan, Australia; 2 Hunter Medical Research Institute, Newcastle, Lot 1 Kookaburra Circuit, New Lambton Heights, NSW 2305 Australia; 3 The Queensland Brain Institute, The University of Queensland, St Lucia, Australia; 4 School of Education, University of Queensland, St Lucia, Australia; 5 School of Psychology, University of Queensland, St Lucia, Australia; 6 School of Medicine, University of Newcastle, Callaghan, Australia; 7 Schools of Psychological Sciences & Medicine, University of Newcastle, Callaghan, Australia

**Keywords:** Imitation, Counter-imitation, Heirachical Gaussian filter, Rescorla–Wagner, Learning, Reaction time, Bayesian active inference, Predictive coding

## Abstract

Dyadic interactions require dynamic correspondence between one’s own movements and those of the other agent. This mapping is largely viewed as imitative, with the behavioural hallmark being a reaction-time cost for mismatched actions. Yet the complex motor patterns humans enact together extend beyond direct-matching, varying adaptively between imitation, complementary movements, and counter-imitation. Optimal behaviour requires an agent to predict not only what is likely to be observed but also how that observed action will relate to their own motor planning. In 28 healthy adults, we examined imitation and counter-imitation in a task that varied the likelihood of stimulus–response congruence from highly predictable, to moderately predictable, to unpredictable. To gain mechanistic insights into the statistical learning of stimulus–response compatibility, we compared two computational models of behaviour: (1) a classic fixed learning-rate model (Rescorla–Wagner reinforcement [RW]) and (2) a hierarchical model of perceptual-behavioural processes in which the learning rate adapts to the inferred environmental volatility (hierarchical Gaussian filter [HGF]). Though more complex and hence penalized by model selection, the HGF provided a more likely model of the participants’ behaviour. Matching motor responses were only primed (faster) in the most experimentally volatile context. This bias was reversed so that mismatched actions were primed when beliefs about volatility were lower. Inferential statistics indicated that matching responses were only primed in unpredictable contexts when stimuli–response congruence was at 50:50 chance. Outside of these unpredictable blocks the classic stimulus–response compatibility effect was reversed: Incongruent responses were faster than congruent ones. We show that hierarchical Bayesian learning of environmental statistics may underlie response priming during dyadic interactions.

**Supplementary Information:**

The online version contains supplementary material available at 10.3758/s13423-022-02161-6.

Interacting with another person is an essential human experience, resting upon the integration of the perception and execution of actions. During action observation, sensorimotor integration is underpinned by “mirroring” subserved by a network of frontoparietal cortical regions dubbed the mirror neuron system (recent review: Heyes & Catmur, 2022; Molenberghs et al., 2012; Rizzolatti & Craighero, 2004). Action mirroring develops through associative learning of stimulus–response (SR) relationships, which is biased towards “mirror-matched” actions and leads to a tendency towards imitation (Catmur et al., 2007; Catmur et al., 2009). Yet, this tendency can be modulated according to context and experience (Bardi et al., 2015; Campbell et al., 2018; Catmur et al., 2007; Catmur et al., 2018; Cook et al., 2012). The classic behavioural marker of mirroring is a kind of stimulus–response compatibility (SRC) effect: a reaction-time mismatch cost with faster responses to stimuli that show the same action as the response action and slower reactions to incompatible actions (Heyes, 2011). Training to perform incompatible responses can lead to a reduction or reversal of mismatch costs (Bardi et al., 2015; Catmur et al., 2008; Cavallo et al., 2014; Heyes et al., 2005). Recent work has shown that even brief observation-execution training with unfamiliar gestures can modulate the activity in sensorimotor cortices during subsequent action observations (Brunsdon et al., 2020), suggesting that short-term training has effects on “mirror” representations.

Anticipating the future actions of others, to in turn prepare one's own actions, is essential for fluid interpersonal interactions. One way to achieve adaptive sensorimotor encoding of observed actions is by learning through predictive coding and active inference (Friston et al., 2009; Friston & Kiebel, 2009; for further commentary: Clark, 2013, for a review of predictive coding algorithms: Spratling, 2017). Predictive coding posits that the brain does not passively receive input but rather is an active system comparing a prediction of upcoming sensory information (top down) with new inputs (bottom up), and efficiently processing this information by only relaying the difference between the prediction and sensation, termed the *prediction error* (Rao & Ballard, 1998). Prediction errors can be weighted by the estimated precision of different sources of information (Moran et al., 2013; Yon & Frith, 2021). Less precise prior beliefs allow for a greater degree of uncertainty about external causes of sensations. As a result, the weight afforded to new inputs is lessened relative to top-down predictions when sensory information is noisy. Conversely, when sensory inputs are precise, beliefs should be updated to suit this new information. Furthermore, if one believes that the environment is volatile, holding precise prior beliefs based on recent experience carries a risk of high prediction errors. This higher-order estimate of uncertainty—describing the volatility of the environment—allows the rate of belief-updating to adjust to an ever-changing world (Yon & Frith, 2021).

Relevant to action execution/observation is that these perceptual inference models link sensory and motor processes (Kilner et al., 2007a, b; Körding & Wolpert, 2004). Anticipatory preparation of response actions is facilitated when one’s sensory predictions are precise and reliable (Behrens et al., 2007; Mathys et al., 2011). Predictive coding accounts have been established for action execution (Adams et al., 2013; Gale et al., 2021; Körding & Wolpert, 2004; Wolpert et al., 2011; Wolpert & Flanagan, 2001), action observation (Urgen & Saygin, 2020), and action mirroring (Kilner et al., 2007a, 2007b; Schippers & Keysers, 2011). The generative model used to predict the sensory (proprioceptive and visual) effects of one’s own movements can be adapted to also predict the sensory (visual) effects of someone else’s actions (Kilner et al., 2007a). By extension, such inferences about the kinematic causes of an observed action allows for the observer to map out their own motor plans for imitation (Kilner et al., 2007b), or indeed counter-imitation. Both simulations (Friston et al., 2011) and behavioural data (Neal & Kilner, 2010) have validated predictive coding account of mirroring. Still, it remains unclear whether such sensorimotor predictions can be leveraged flexibly to adapt SR-mapping contextually. In an instance where the imperative stimulus is an action (as in classic automatic imitation tasks) it becomes necessary to not only predict what action will be observed but also how that observed action might relate to your own motor planning. A reliable prediction that a stimulus-action will not match a response-action should allow for preparatory control of mirror-matched or stimulus congruent action representations (Cross & Iacoboni, 2014; Campbell & Cunnington, 2017) and reduce reaction-time mismatch costs.

To prepare optimal behaviours, an agent must learn the association between stimulus and response. Under classic reinforcement learning models, the belief that given events are associated (SR pairings) should strengthen with repetition. Prediction errors are steadily reduced with each instance of expected SRs, increasing the associative strength between them (Sutton & Barto, 2018). The Rescorla–Wagner (RW) model of reinforcement learning (Rescorla & Wagner, 1972) was developed to account for this effect and does so in a relatively simple manner. RW models offer computational efficiency and have also shown great utility in functional neuroimaging studies of learning (Wang et al., 2016). However, RW assumes that the rate of learning is constant, which may be suboptimal for any environment that changes rapidly (Behrens et al., 2007). Under Bayesian learning accounts, the learning rate should be a function of the agent’s uncertainty (Behrens et al., 2007). Extending on this, the hierarchical Gaussian filter (HGF; Mathys et al., 2014; Mathys et al., 2011) models learning as a multilevel generative model, where the learning rate is adjusted by different sources of uncertainty, as well as subject-specific parameters that can reflect individual differences in how rapidly beliefs are updated.

Here, we address whether statistical learning of SR-pairing modulates behaviour during an action observation-execution task. We modified an SRC action task (Brass et al., 2000; Brass et al., 2001; Brass et al., 2009; Campbell et al., 2018; Cross & Iacoboni, 2014; Heyes, 2011; Press et al., 2008) to study the influence of the predicted SR relationship on motor preparation. We manipulated the likelihood of SRs being congruent or incongruent, and by extension the likelihood that the mirrored representation of a stimulus would facilitate or interfere with the execution of one’s own action. Critically, prior studies have matched the likelihood of SR match and mismatch, making both pairings unpredictable (probability of SR congruence = 0.5). To our knowledge, the effect of probabilistic manipulations of uncertainty (and volatility) on mirroring behaviour have not been tested. We used computational models (the RW and HGF models) to test the hypotheses that not only do humans learn the likelihood of mirror-matched SR pairings (probability context), but that the effects of SRC can be controlled, so that reaction-time mismatch costs can be reduced or reversed. Comparing evidence for the RW versus the HGF model allows us to investigate whether a constant learning rate is enough to optimize responses (RW model) or whether a variable learning rate allows adaptation to uncertainty of sensorimotor inputs as well as their volatility (HGF model).

## Methods

### Participants

Experimental data were acquired from 31 healthy, right-handed participants (mean age = 20.8 years, range: 18 to 25 years, 20 females, and mean handedness score 0.95, *SD* = 0.11). Data from three participants were excluded: two due to failing to adequately performing the task (20% and 26% of trials missing response time data), and a third participant due to technical errors (only 6 of 10 blocks were run). This left a final sample of 28 (18 females, mean age = 20.7 years, range: 18 to 25 years, mean handedness score = 0.95, *SD* = 0.12).

A priori power estimation using G*Power (Faul et al., 2009), based on performance on our previous behavioural task and the observed main effect of congruence with partial η^2^ = 0.234 (Campbell et al., 2018), we expected an effect size *f* = 0.553. Aiming for power of 0.90 the estimated minimum sample-size was eight participants. Given this provided a lower-limit minimum to replicate our previous mismatch-cost findings for this kind of stimuli, we were confident of a final sample size of *n* = 28 being sufficiently powered for this first attempt at probabilistic manipulations of SRC. Note that this a priori power analysis was based on a simpler paradigm (four conditions in 2 × 2 factorial with the same stimulus–response action pairs), so to accommodate for the additional complexity of the current paradigm and analysis plan, including trial-wise modelling, we targeted a sample of 30 participants. This sample also aligns with the sample size used in other multifaceted SRC tasks (e.g., Ainley et al., 2014).

### Behavioural Task

Participants performed a variation of our previous SRC action task (Campbell et al., 2018). Behavioural and autonomic responses were recorded while experimental stimuli were presented with custom MATLAB (Version 2018b, The MathWorks, Natick, MA) scripts run with the Psychtoolbox extensions (Brainard, 1997).

**Fig. 1:**
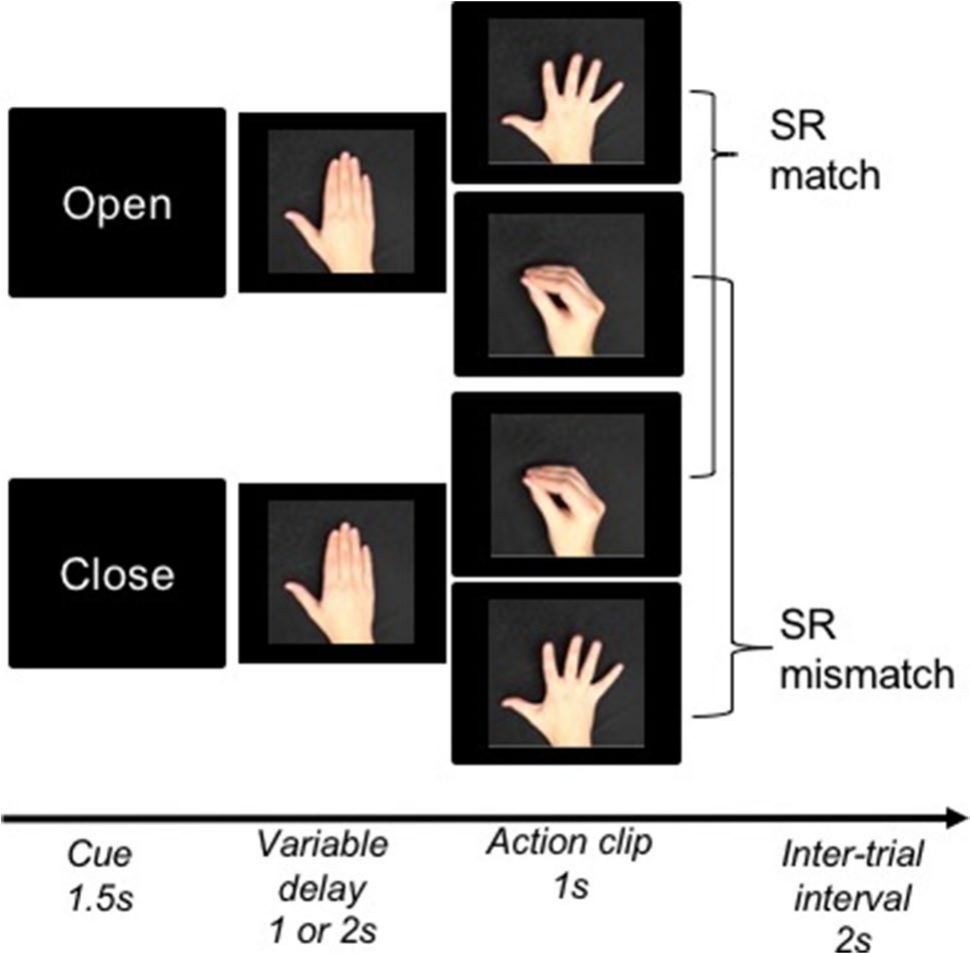
Behavioural paradigm showing manipulation of the SR (SR) congruence with diagram of the task trials, with all combinations of cues and stimuli depicted, producing matching or mismatching SR pairs and the timing of trial events. The final frames of example stimulus videos are shown

**Fig. 2:**
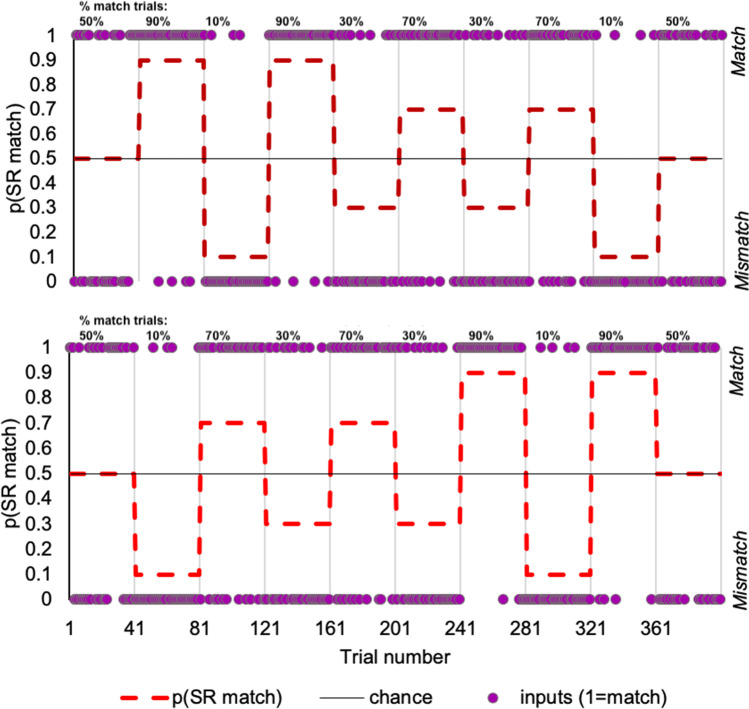
Example sequences of 400 trial across 10 blocks for the behavioural task, demonstrating our block-wise manipulation of the likelihood of SR congruence. The order of changing likelihoods (red dotted line, plotted as the probability of a matching SR pair) was counterbalanced across participants with two alternative sequences (compare the top and bottom plots show block orders 1 and 2). Within each block a random order of trials was generated for each participant. Example runs of trial types for each block sequence are displayed in purple (1= SR match, 0= mismatch), and this served as a binary input for computational models. (Colour figure online)

Participants performed an action execution/observation task in which they were cued to prepare either an opening or closing action with their right hand and then perform this action while watching a video of a hand gesture that *incidentally* matched or mismatched their planned action (Fig. 1). The likelihood of SR congruence (ratio of match:mismatch trials within a block) changed across five levels such that the probability of a match trial was either 0.9, 0.7, 0.5, 0.3, or .01 (Fig. 2), and so produced a 2 (SR congruence) × 5 (likelihood of SR congruence) factorial design.

The crucial manipulation was the likelihood of SR congruence. Importantly, the ratio of the two stimulus-types (“open” or “close” action video) and the two action cues was maintained at 50:50 throughout the task. This ensured that before the cue was presented, the a priori probability of the stimulus video being an open or close video was 50%. Hence, any expectations about the stimulus could only be relative to the cued response (i.e., the expected SR congruence of cued and observed actions for a that particular trial; den Ouden et al., 2010).

The likelihood ratio for match:mismatch was either highly predictable (0.9 or 0.1), moderately predictable (0.7 or 0.3) or unpredictable (0.5). This “hidden” probability changed across 10 blocks of 40 trials as the participants were not informed of the timing or range of probability changes. Given the shifting probabilities across trial-blocks, there was a changing expectancy or degree of “surprise” for trial-wise SR congruence. It is important to note that creating these changing probabilities did necessarily limit the number of the least likely trial types. With 40 trials per block (10 blocks, totalling to 400 trials), the trial number ratios of match:mismatch were: 36:4, 28:12, 20:20, 12:28, 4:36. As each block type occurred twice, the total trial numbers for each block type were 72:8, 56:24, 40:40, 24:56, 8:72.

Two block orders were varied across participants to ensure the robustness of our manipulation to sequence effects (Fig. 2). A block with 70% of trials matching SR pairs, and 30% mismatching is referred to as a 0.7 block, while the opposite ratio of match to mismatch is referred to as a 0.3 block. In both possible sequences, the steps between consecutive blocks involved a shift of at least 40% likelihoods; each context occurred twice and the first and last blocks were both 0.5 contexts (the second block sequence was the first sequence in reverse order).

#### Procedure

Participants were seated approximately 60 cm from the visual display and began each trial by resting their right hand on the space-bar key in preparation to perform a right-handed gesture, with movement onset marked by the key release. A word cue indicated one of two opposing actions—opening or closing hand gesture—and participants were instructed to execute this gesture at the onset of movement stimulus. Participants were instructed to “Respond as quickly, but as accurately as possible once the on-screen hand moves. You have to see the movement but do not have to wait for it to finish.” Reaction time was calculated as the time between the onset of the onscreen movement (imperative stimulus) and the participant releasing the key. The movement stimuli were 1-s clips of a hand performing either the opening or closing action. The 1-s duration of this stimulus was also the response period, during which a participants reaction time was recorded by the release of the space-bar key. Responses beyond this period were not recorded. Clips of both a male and female actors’ hands were included and were presented in counterbalanced order across trials. A static hand in the resting position was displayed between the word cue and stimulus movement with a variable delay of 1–2 s. This ensured that participants could not anticipate the precise onset of the movement stimulus. During the intertrial interval (2 s of central fixation), participants held the space key and rested their hand in neutral position. While conducting the experiment, the experimenter observed action execution to confirm accurate performance was maintained.

### Data Analysis

#### Behavioural Analysis

We examined SR congruence in terms of mean reaction time, which was used to calculate reaction-time mismatch costs (difference between mean for match and for mismatch trials) for each probability context. The manipulation of SR-match probabilities resulted in uneven trial numbers, with the least likely trials being too few to estimate the mean reaction-time costs. Specifically, only eight trials occurred for each of the two least-likely trial types (a match within 0.1 blocks and a mismatch within 0.9 blocks), so we limited analyses of the reaction-time mismatch-cost effect to the blocks with p(SR match) of 0.3, 0.5, 0.7. The main effect of probability context (across these three levels) on mean reaction-time difference was subject to a nonparametric repeated measures test (Friedman chi-squared with Conover post hoc test for pairwise comparisons with Bonferroni correction). To formally assess the likelihood of any mismatch cost effect against the null, we additionally used a Bayesian analysis of variance (ANOVA), conducted in JASP (JASP Team, 2020; Rouder et al., 2017; Raftery, 1995).

Failure to respond within the response period (i.e., within the duration of 1-s movement stimulus), either responding too early (anticipating the movie) or not responding within 1 s was classified as a missing data point. For the 30 participants who completed 10 blocks of the task, the mean percentage of missing trials by condition are presented in the Supplementary Material Table S1a, with Table S1b showing this for the analysis sample of 28 participants.

#### Computational Modelling

Trial-by-trial learning models were compared to examine whether static reinforcement (the Rescorla–Wagner model [RW]) or dynamic and hierarchical learning (the hierarchical Gaussian filter model [HGF]) better explain trial-wise variations in reaction times in our data. Here, we hope to provide a conceptual overview of the HGF, and point interested readers to the detailed descriptions outlined by Mathys et al. (2014; Mathys et al., 2011), as well as the documentation within the HGF toolbox (Frässle et al., 2021; translationalneuromodeling.github.io/tapas/).

The HGF models an agent’s belief about external causes of the sensory inputs they experience (perceptual model), paired with a response model of the behavioural consequence of these beliefs. For binary stimuli (as in our paradigm), the lowest level predicts the most likely input between the two alternative events (SR match or mismatch events). This low-level prediction is informed by higher-order predictions of how likely either event is given previous experience (the current tendency towards SR matching), and how stable this tendency is over time. These beliefs are captured as distributions, with states evolving over time as Gaussian random walks, with the step-size (variance) for each level informed by the next level above. These distributions can thus be described by their sufficient statistics (mean and variance), with the mean representing the current prediction for that event/tendency, and the variance reflecting the uncertainty (inverse of precision) for that prediction. Evolving beliefs about multiples sources of uncertainty in the agent’s model of the environment are also captured within the corresponding level of the hierarchy. The prediction error at the first level is simply the difference between predicted and incoming information, weighted by the precision of the agent’s current sensory beliefs. At higher levels of the hierarchy, the precision captures uncertainty about “expected uncertainty,” with associated prediction errors weighting the updates of these higher-order beliefs. It is this precision-weighting of prediction errors passing up the levels of the HGF that allows for the learning rates to be adjusted adaptively. Belief-updates at a given level of the model will be determined by a combination of the previous level’s precision-weighted prediction-error, as well as subject-specific parameters that govern how uncertainty influences learning for that individual (omega and theta). These parameters determine the coupling of each level to the previous, that is how lower-level states are influenced by higher-level states. Theta (ϑ) captures subject-specific estimates of “meta-volatility”—the agent’s uncertainty about the current degree of environmental stability, given past experience of changes (*phasic* volatility). If an agent is confident in their prior estimates of environmental uncertainty and expects little to change, their learning rate is slowed, and this phasic volatility will be low. Omega (ω) captures a constant component of volatility, the *tonic* volatility, and determines how quickly individuals update their beliefs about environmental contingences in general. Together, these control the speed of belief-updating at Levels 2 and 3, in a three-level HGF.

**Fig. 3:**
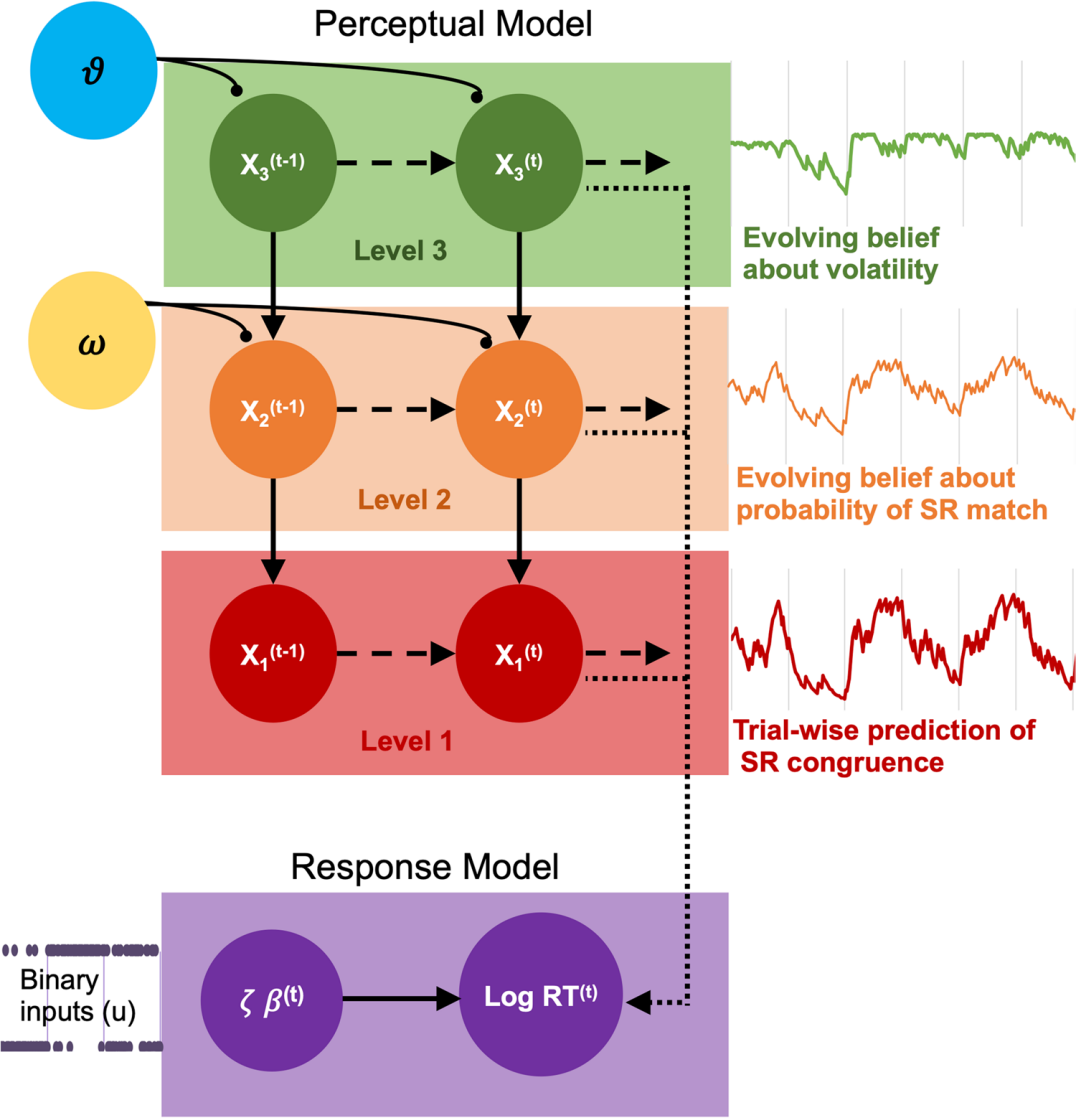
Representation of the perceptual and response models of the HGF (adapted from Marshall et al., 2016). Beliefs are represented in probability distributions arranged hierarchically, with the updating of each level influenced by the estimate in the level above. The perceptual model tracks the participant’s learning of task structure in three levels: the trial-wise encoding of SR pairs (x_1_), the probability of SR congruence (x_2_) and the volatility of this tendency over time (x_3_), for the current trial *t*. Omega and theta (ω, ϑ) are parameters that couple the levels and control the rate of belief updating for that participant. The response model maps the participant's trial-wise beliefs onto the observed changes in log reaction time (RT), with decision noise captured by zeta (ζ, Gaussian noise term). (Colour figure online)

Within the current task, the generative model at the lowest level, a trial-wise prediction about the SRC of an impending event is updated given the most recent input. Any prediction errors are propagated up the hierarchy and compared with the predictions generated at each level above (Fig. 3): Level 1 produces sensory prediction errors, and at Level 2, prediction errors for the likelihood of a SR match, and Level 3, prediction errors for the beliefs of the stability of these likelihood contexts (Vossel et al., 2014, Fig. 3, red, orange, and green panels respectively).

Our perceptual and response model parameters were estimated from trial-by-trial binary inputs (match or mismatch) and log reaction times (Bröker et al., 2019, Fig. 3; Marshall et al., 2016). The initial priors for the model parameters were based on a model with only the binary experimental inputs (trial types: match or mismatch), without any observed responses. The optimal model parameters were then estimated through variational Bayes (Table 1).

**Table 1:** Mean and variance of Gaussian priors used in parameter estimation for the hierarchical Gaussian filter perceptual and response models

HGF model	Parameter	Prior mean	Prior variance
*Perceptual*	Tonic volatility (ω) (Level 2, Level 3)	−4, −2	1, 1
*Response*	Decision noise (Log σ ζ)	1.0972	0.6931
	Baseline logRT (β0)	6.2146	4

“Tapas_hgf_binary_config.m” for setting perceptual model priors, and for response model priors defaults as per “tapas_logrt_linear_binary_config.m.”

For comparison, we also modelled trial-wise learning with the RW model (Rescorla & Wagner, 1972). The RW model describes expectation-outcome associations through reinforcement learning in the presence of a stable (constant) learning rate and without hierarchical representation of beliefs. As with the HGF, the RW model also estimates prediction error as the difference between the expected and observed outcome for that trial. This is used to update expectations for the next trial, weighted by a fixed learning rate, which can be used to predict trial-wise reaction times (Jones et al., 2011). The RW model implies that the precision of prediction errors do not vary trial-to-trial and do not adjust to the agent’s estimate of environmental volatility. It is thus less flexible, yet more parsimonious than the HGF.

We implemented the following families of functions from the HGF Toolbox (Version 5.3; http://www.translationalneuromodeling.org/tapas/): HGF perceptual model and response model respectively, with the “tapas_hgf_binary” (Mathys et al., 2011), and “tapas_logRT_linear_binary” (Marshall et al., 2016); with the optimization algorithm “tapas_quasinewton_optim”; for RW model “tapas_rw_binary” and “tapas_gaussian_obs” (for continuous responses).

To model reaction times, it is necessary to couple the perceptual model (RW or HGF) to an appropriate response model. As a proof of concept, we have augmented the perceptual model with a simplified drift-diffusion decision model (Ratcliff et al., 2016). Our model approximates the response time distributions using a Wald (or inverse Gaussian) model, which is the marginal distribution of first-passage times for a continuous random walk (Brownian motion) through a barrier. This represents a classic decision model, whereby the likelihood of a motor decision accumulates according to current evidence, influenced by prior beliefs about the outcome. The inputs for this response model for the RW model were the trial-wise priors (beliefs) about the current trial being a match/mismatch as per the RW static learning rate. The inputs for the HGF were a linear combination of the Level 1 parameter (the posterior expectation of the probability of a match trial) and the Level 3 parameter (the dynamic estimate of volatility in the probability of match trials).

Parameters for the response model included: the mean drift rate, a minimum offset from zero for a reasonable reaction time (0.2 seconds) and, a decision model accumulator to link the perceptual parameter values. The threshold for a response was fixed arbitrarily at 1, to constrain a scaling property of the model. Initial values for the mean drift rate parameter and the decision model accumulator were set 6 and 1. These values were then optimized iteratively using the group-mean values. Subject-wise optimized mean drift diffusion and decision accumulator were then estimated by optimizing a log likelihood function over subject-wise reaction-time distributions. Once optimized per participant, the drift-diffusion response model was then simulated to estimate trial-wise RT (in seconds). In addition to showing exemplar trial-wise reaction times, we also examined the estimation of subject-wise mean RT and group-wise condition-specific mean RT.

#### Bayesian Model Selection

To test which model (HGF vs. RW) was best able to explain our behavioural data, we implemented Bayesian model selection (BMS) in the VBA toolbox (Daunizeau et al., 2014; Rigoux et al., 2014; Stephan et al., 2009). Accordingly, the log model-evidence (LME) for the two perceptual models was calculated for each subject using variational Bayes. The relative evidence of alternative models was then compared, balancing model accuracy against complexity (Rigoux et al., 2014; Stephan et al., 2009; Raftery, 1995): The more complex HGF compared with the RW model is thus penalized for its additional parametrization. We compared the exceedance probability of our two computational hypotheses: (1) dynamic learning rates informed by multiple levels of beliefs about uncertainty (the HGF approach), and (2) a stable reinforcement learning rate embodying cumulative experience from preceding trials (the RW approach). The exceedance probability is the probability that, given the data, a particular model is more likely than the other model.

To compliment this group-level BMS, we also considered LME at the participant level. Taking the difference between each participant’s LME for the HGF versus the RW, we described the model selection for each participant, with positive differences indicating the HGF outperforming the RW for that participant, and negative differences vice versa.

## Results

### SR Congruency Reaction-Time Effects

**Fig. 4:**
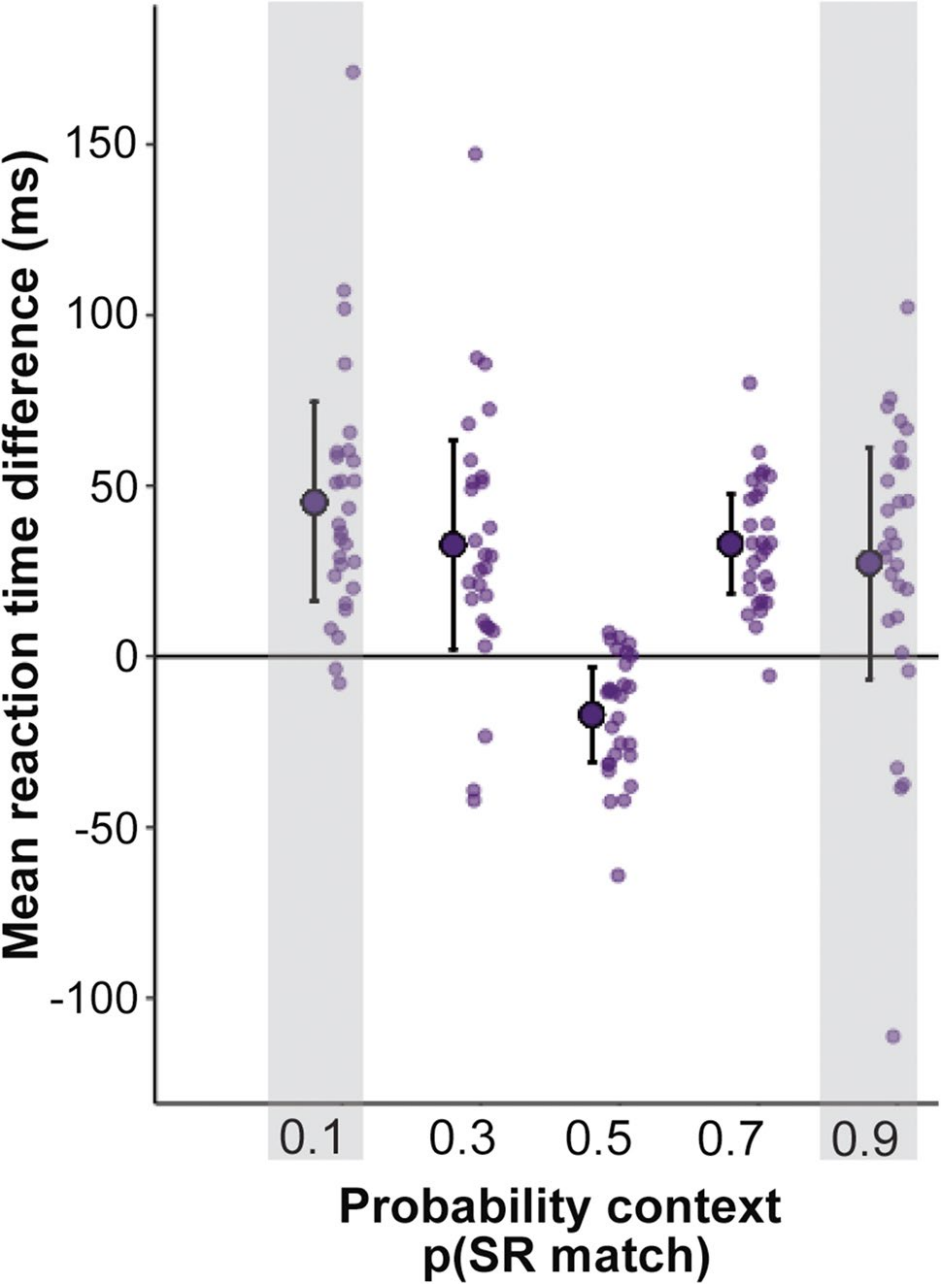
Mean reaction-time difference for match–mismatch, with negative values indicating a “mismatch cost.” The dark purple circles and error bars show the group mean with ±95% confidence intervals around the group mean. Transparent dots represent individual participant means. ** denotes Bonferroni-corrected *p* < .001, NS = not significant. *Note*. The greyed shading for the probability contexts of p(SR match) 0.1 and 0.9 indicates that these were not included in the statistical tests for the effect of probability on RT differences due to the fewer number of trials*.* (Colour figure online)

Analysis of the behavioural data with repeated-measures (Friedman chi-squared) test indicated that reaction-time (RT) differences (match-mismatch) were significantly influenced by the likelihood of an SR match, χ^2^(2, *n* = 28) = 39.5, *p* < .001, Kendall’s *W* = 0.496 (moderate effect size; Fig. 4). Pairwise comparisons showed that the reaction-time cost present in the block with p(SR match) of 0.5 was significantly different from that in both the 0.3 and 0.7 blocks (both *p*s < .001; Table 2 provides details of the Conover’s post hoc test, and Table 3 provides mean differences in reaction times for all probability conditions). A Bayesian repeated-measures ANOVA test for this same main effect of probability on the RT across 0.3, 0.5, and 0.7 blocks, indicated that a model including the effect of probability was far more likely to account for the observed RT differences than a null model, p(M1 | data) = 1 versus p(M null | data) = 9.958E-11, BFM1= 1.004E+10 (Raftery, 1995). Mismatch costs in the rarer conditions (0.1 and 0.9 blocks) showed the same qualitative effects, although we did not include these in the statistical inference owing to their small number in each subject.

**Table 2:** Conover’s post hoc comparisons for mean reaction-time differences (match–mismatch) across three probability conditions: 0.3, 0.5, 0.7

p(SR match) blocks		*T*-Stat	*df*	W _i_	W _j_	p _bonf_	p _holm_
0.5 Vs	0.3	5.078	54	67	29	<.001	<.001
0.5 Vs	0.7	5.746	54	29	72	<.001	<.001
0.3 Vs	0.7	0.668	54	67	72	1	.507

**Table 3:** Descriptive statistics for the “mismatch cost” reaction-time difference for match–mismatch, by probability context

Probability Context P(SR match)	Reaction-time difference (ms) for match–mismatch			
	Mean	*SE*	Median	IQR
**0.1**	45.218	7.113	37.520	36.107
**0.3**	32.696	7.421	27.640	41.477
**0.5**	−16.958	3.381	−11.000	27.938
**0.7**	32.975	3.556	32.350	28.660
**0.9**	27.403	8.25	32.370	45.460

IQR = Interquartile range. Greyed rows indicates that for p(SR match) conditions of 0.1 and 0.9, the least likely trial types are too few to estimate the mean reaction time accurately (eight trials each)

### Bayesian Model Selection (BMS) of HGF Versus RW Models

**Fig. 5:**
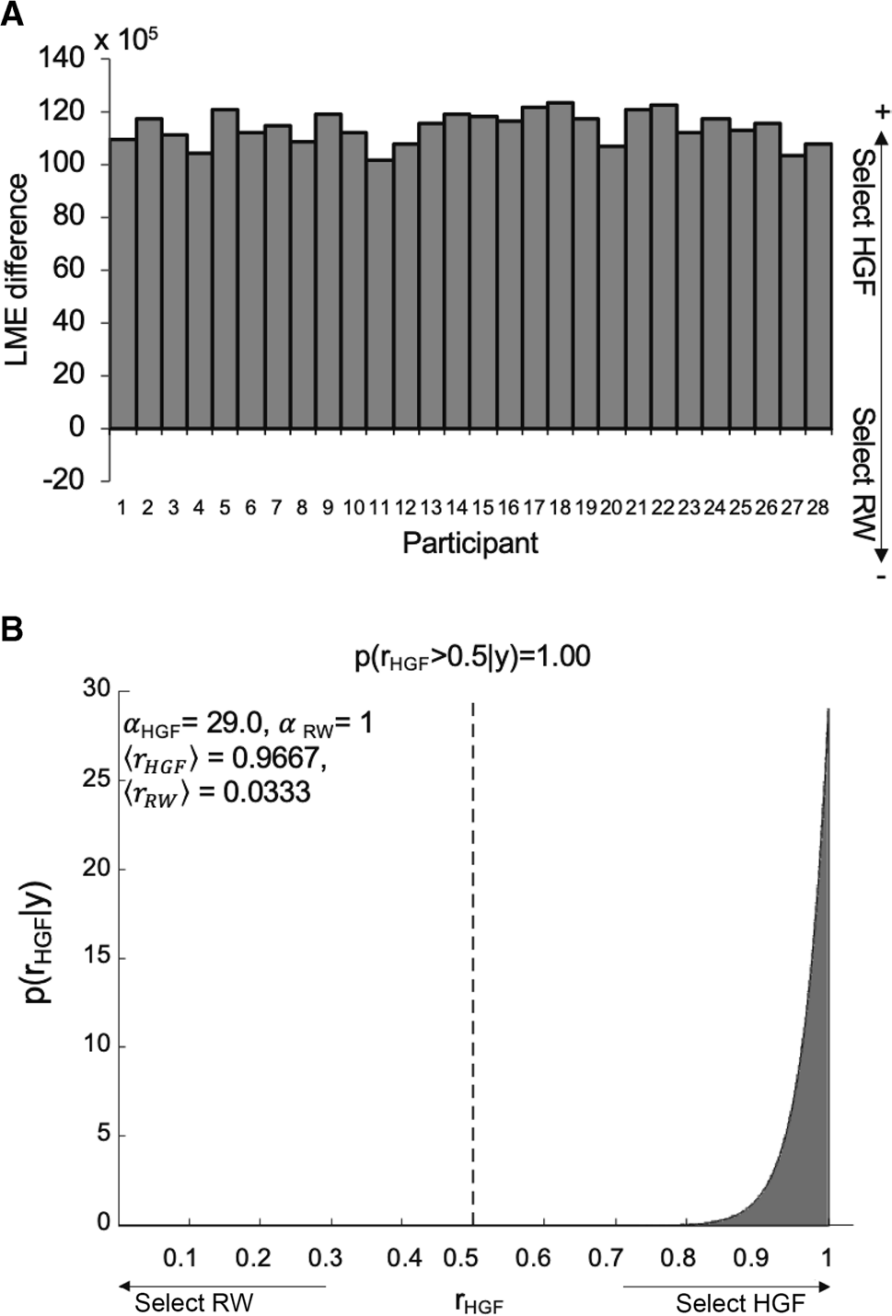
**A** Difference in log model evidence (LME) for HGF and RW models for each participant. Positive values indicate model evidence favours the HGF. **B** Dirichlet density describing the probability of model 1 (HGF model) given the data y (log RT). The shaded area representing the exceedance probability of the HGF being more likely than the Rescorla–Wagner model; variational Bayes estimates of the Dirichlet parameters of each model: α_HGF_ = 29, α_RW_ = 1;〈r〉conditional expectations of the probabilities of the two models

At the participant level the root-mean square error (RMSE) for the HGF was consistently much lower than that for the RW model. For the HGF, the RMSE ranged from 0.175 to 0.363 as compared with the RW model with a range of 5.099 to 5.773, implying that the HGF was better able to predict the observed data. Formal model comparison showed that the HGF was still favoured over the RW alternative (after taking into account its extra complexity) for all 28 participants, indicated by positive LME differences (Fig. 5a).

BMS at the group level indicated that the HGF model outperformed the RW model, with the protected exceedance probability (pxp) for the HGF approaching 100% (compared with the RW pxp = 5.588 × 10^-8^). This pxp of >99% (Bayesian omnibus risk BOR of 1.080 × 10^-7^), indicates strong evidence for the HGF over the RW model (Rigoux et al., 2014), again after accounting for its greater complexity. The values for conditional expectations of model probabilities also reflected this result, with 96.7% for the HGF versus 3.33% for the RW, further confirmed by the Dirichlet density for the relative probabilities of the models (Fig. 5b).

### HGF Modelling of Responses to Variable SR Congruence

**Fig. 6:**
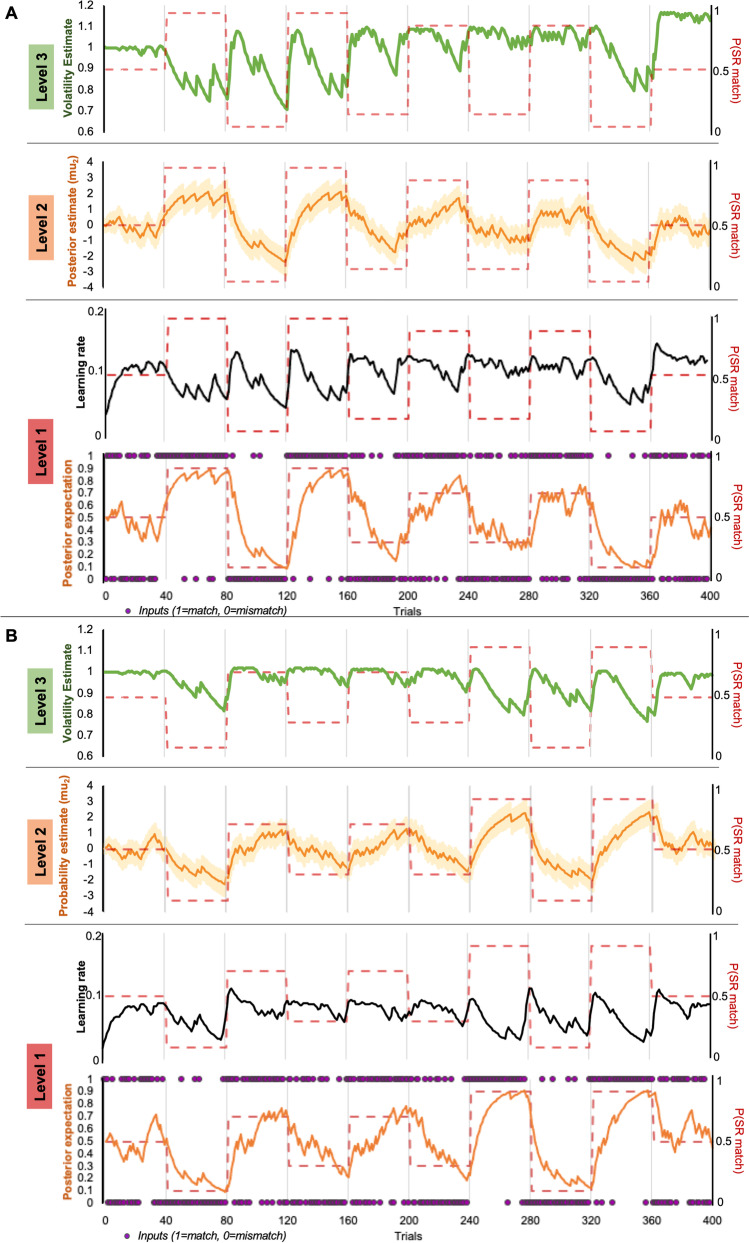
Two example trial-by-trial trajectories of HGF estimates for each block sequences (**A** and **B**) given binary input (purple dots, Level 1) and block-wise probability of match trial (dashed red lines, Level 1) of expected input (orange), learning rate (black); Level 2: tendency (yellow) volatility (green, Level 3). (Colour figure online)

The HGF qualitatively followed reaction-time differences across the alternating sequences of blocks (see two example participants, Fig. 6). The learning rate for both participants (thin black line in Level 1) consistently peaks shortly following the changes in the underlying (true) SR likelihood (dashed orange line plotted at Level 1). Accordingly, the posterior estimates of SR likelihood at Level 2 adjust to approximate the true probability shifts. This implies that participants adjust their behaviour according to the shifting contexts, implicitly learning the underlying (true) SR likelihood.

Within each (hidden) block, the expectations about likelihood and volatility are appropriately stable and hence so is the learning rate. Estimates of volatility (Level 3) dip in the highly predictable blocks (0.9 and 0.1 contexts), compared with the moderate and uncertain contexts (0.7, 0.3, and 0.5). In a predictable block, an unexpected trial type (surprising event) provokes a brief rise in volatility estimates, which then settles following the ensuing confirmatory trials. In contrast, the absence of such sequences in the less predictable blocks ensure that the volatility does not settle down as low.

Increases in the learning rate and volatility following transitions to a new block also highlights how the HGF captures the (true) uncertainty at block transitions. At these transitions, the belief of volatility increases with previous expectations becoming less certain because the environment has changed, and the new context is as yet unclear. This transition then requires an increase in the learning rate to adapt to the new context. In the example participants’ trajectories (Fig. 6), the pattern for both the posterior expectations of trial type (red, Level 1) and the estimates of SR likelihoods (Level 2) do indeed track with the true changes in probability, across both block sequences.

**Fig. 7:**
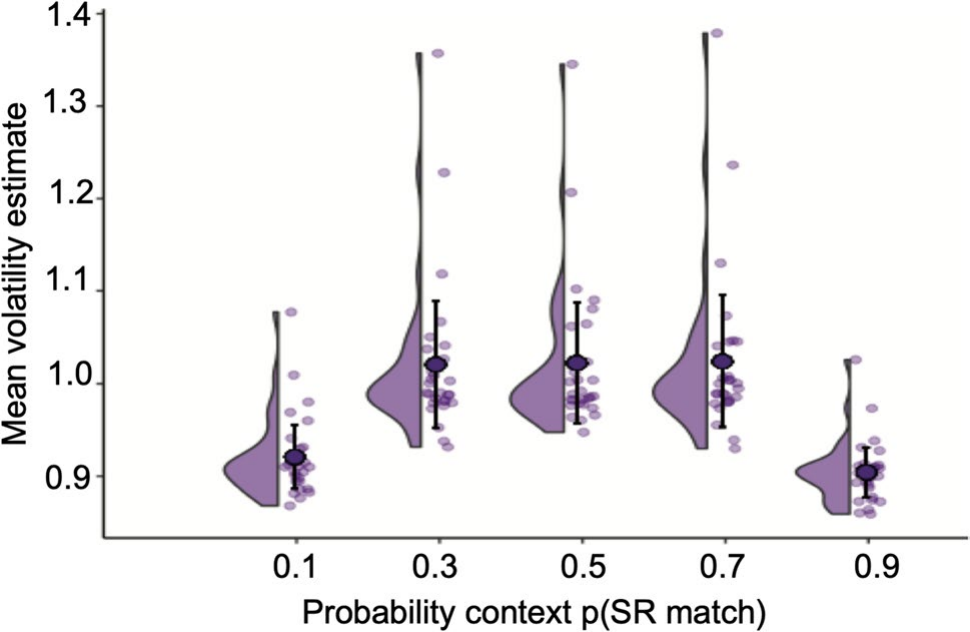
Mean and distribution of volatility estimates by block condition (probability context) with error bars showing 95% CI, overlayed on individual subject’s mean volatility estimate

To formally quantify this pattern of changing block-wise volatility estimates, we conducted a post hoc analysis of the group-means across the five block types. A one-way ANOVA showed a main effect for probability of SR match, Greenhouse–Geisser adjusted *F*(31.451, 1.165) = 48.363, *p* < .001, partial η^2^ = 0.642. This was clearly driven by the mean volatility estimates for the two most predictable blocks, p(SR match) 0.1 and 0.9, being significantly lower than for the p(SR match) 0.3, 0.5 and 0.7 (Table 4, Fig. 7). This result is not surprising yet shows that the volatility estimates are performing as expected, an important sanity check on the model estimation.

**Table 4:** Post hoc comparisons of mean volatility between probability contexts

p(SRmatch) context paired *t* test		Mean difference	*SE*	*t*	*p*_Bonferroni_
**0.1**	**0.3**	**−0.100**	**0.012**	**−8.126**	**<.001**
	**0.5**	**−0.102**	**0.012**	**−8.265**	**<.001**
	**0.7**	**−0.103**	**0.012**	**−8.400**	**<.001**
	0.9	0.017	0.012	1.342	1.000
**0.9**	**0.3**	**0.117**	**0.012**	**9.468**	**<.001**
	**0.5**	**0.118**	**0.012**	**9.606**	**<.001**
	**0.7**	**0.120**	**0.012**	**9.742**	**<.001**
0.3	0.5	−0.002	0.012	−0.139	1.000
	0.7	−0.003	0.012	−0.274	1.000

*P* values adjusted for all 10 comparisons with Bonferroni correction

### Drift-Diffusion Decision Model of Reaction Times

**Fig. 8:**
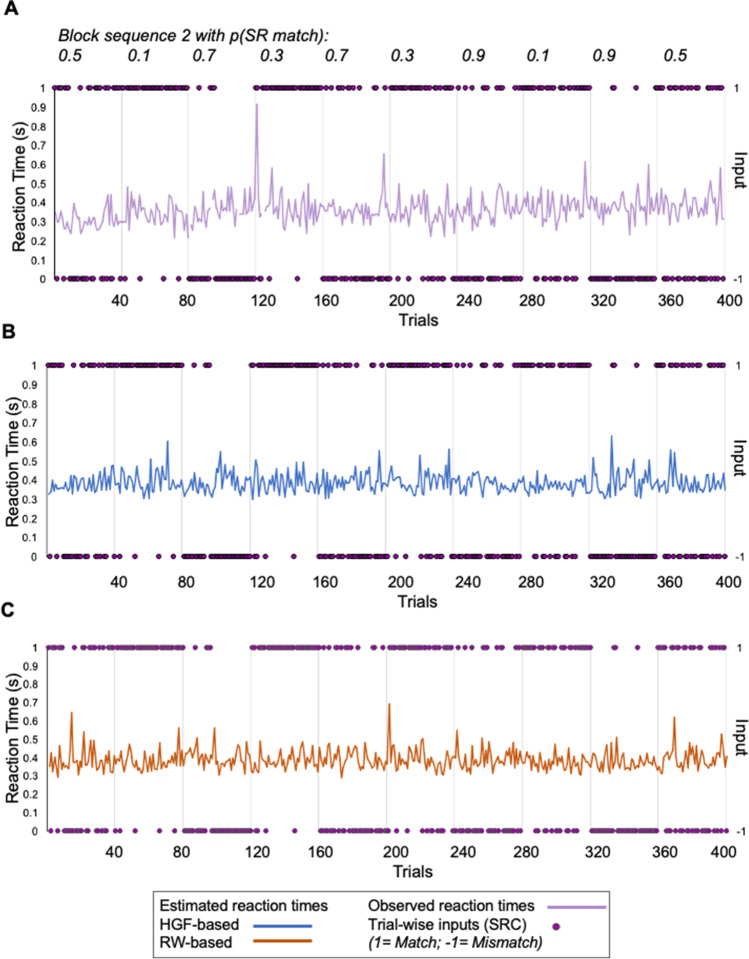
Reaction-time modelling in an example participant. **A** Observed reaction times for a participant completing Block Sequence 2. Predicted reaction times for the (**B**) HGF and (**C**) RW models. (Colour figure online)

**Fig. 9:**
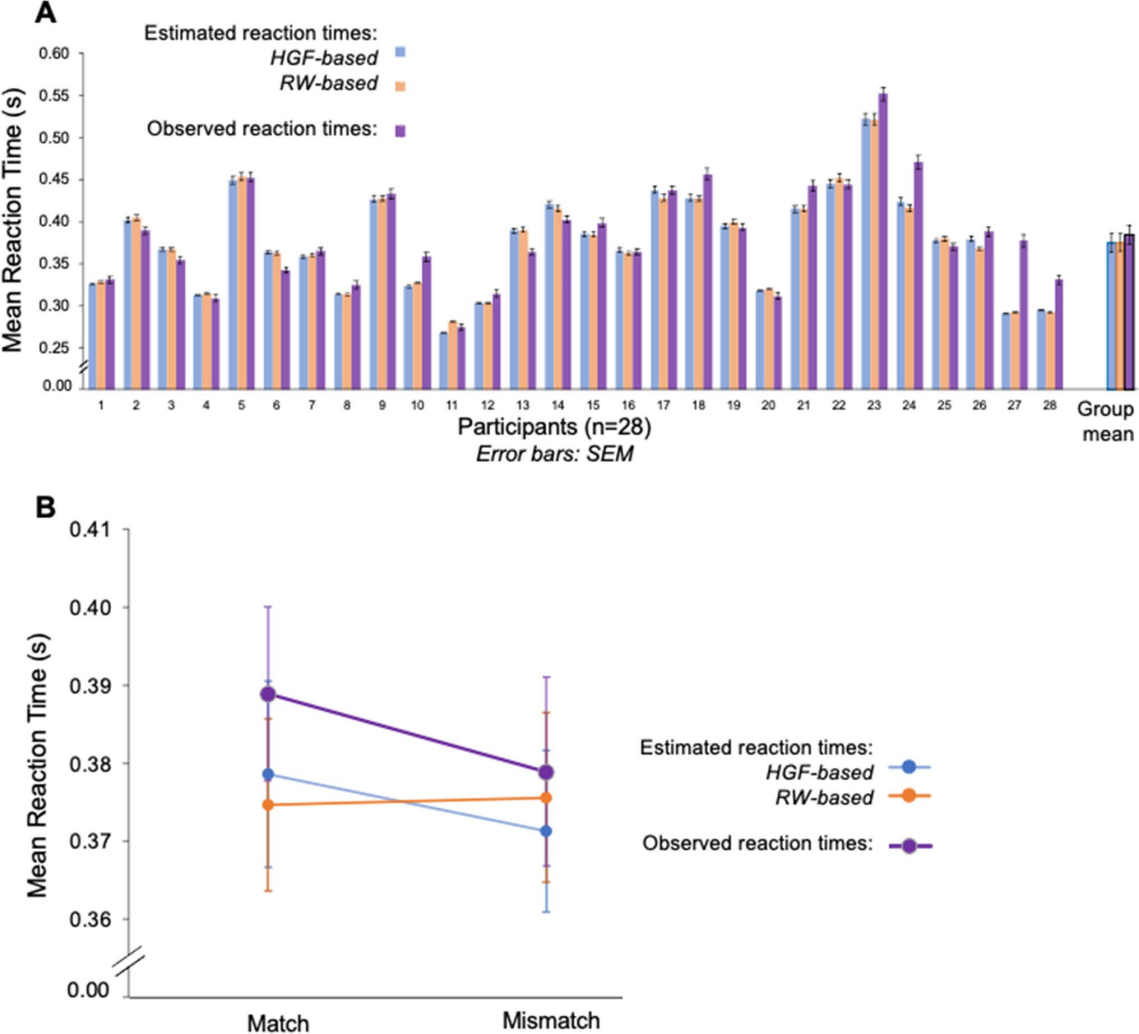
Comparison of estimated and observed mean reaction times using the drift-diffusion model with either HGF or RW perceptual parameters. **A** Subject-wise mean estimates of RT from the diffusion decision models based on either the RW (orange) and the HGF (blue) against the observed (purple) mean reaction time. Overall group mean estimated versus observed reaction time is plotted at the end. **B** Mean reaction times for SR compatibility with observed (purple), against the estimated values for response models with parameters derived from the HGF (blue), and RW (orange) perceptual models. (Colour figure online)

We used a drift-diffusion response model to transform performance of the perceptual model into predicted reaction times, for each of the RW and HGF models. An example subject-specific observed sequence of reaction times and corresponding predictions derived from the HGF and RW perceptual models are shown in Fig. 8(a–c). Both models capture mean and trial-to-trial variation in RT, although the longer tails of the empirical data (the slow responses) are often underestimated. Both models closely capture subject-specific reaction times (Fig. 9a), a result that is consistent with the use of subject-specific maximum likelihood fitting.

Predicted reaction times are on average slightly shorter than observed (Fig. 9a), due to failure to capture the slow tail of the empirical reaction times in some subjects (Supp. Fig. S1). Notably, however, predicted reaction times derived from the HGF—but not the RW—do capture the overall mismatch cost (Fig. 9b), mean differences between match versus mismatch for the HGF estimated reaction times, 0.007 (seconds), 95% CI [0.012, 0.003], *t*(28) = 3.335, *p* = .0024; mean difference between match and mismatch for RW estimated means: −0.001 (seconds), 95% CI [−0.003, 0.001], *t*(28) = −0.855, *p* = 0.4. This is important, as this feature was not built into the response model, but rather emerges from the ability of the HGF perceptual model to capture dynamic changes in learning rate and volatility. That is, the predicted reaction times produced by the drift diffusion models show that perceptual parameters that incorporate volatility (HGF) are better able to capture aspects of the observed data than if using a perceptual model that has a fixed learning rate (RW). Figure 9b highlights that the HGF-predicted RTs capture the main effect of SRC, while the RW-predicted RTs do not. The main effect of probability context was not captured by either response model.

## Discussion

We conducted a novel stimulus–response compatibility experiment, manipulating the likelihood of incidental SR congruence during motor execution. In doing so, we aimed to test if implicit statistical learning may account for how behaviour is shaped by changing SR pairings, depending on their predictability. Our results support the hypothesis that “automatic” mirroring, as implied by SRC reaction-time effects, is modulated by the context-dependent expectation of the SR (in)congruence. Here, learning the likelihood of SR congruence rests upon accumulating evidence from preceding trials—evidence that allows an estimate of the likelihood that the next stimulus will match or mismatch one’s cued action, in a manner that also incorporates the confidence in this prediction. We find that this learning facilitated participants’ faster responses to mismatching trials in predictable contexts, leading to a reversal of the classic mismatch cost. This is an important example of preparatory inhibition of mirroring as a result of statistical learning. Here, the classic SRC reaction-time effects did not persist outside of the 0.5 context. A decrease or reversal in mismatch costs have been previously observed when participants were trained in counter-imitation (such as in Bardi et al., 2015; Cavallo et al., 2014; Heyes et al., 2005). However, to our knowledge, no other study has reported mismatch benefits emerging implicitly from a task, purely on the basis of statistical learning. From our results, it appears that the tendency toward imitative responses is dependent on the uncertainty of upcoming SR pairs. The variation in mismatch costs for contexts in which mismatches were most likely and predictable (0.1 and 0.3 contexts) aligned with our hypothesis. An unexpected result was this mismatch facilitation (faster responses to mismatch than match trials) also occurring in the mismatch-unlikely contexts—where p(SR match) = 0.7 and 0.9. We speculate that participants may be primed for the mismatch unless the SR pairing is at perfect (50%) chance levels. This prior would be an adaptive way to prepare for the more difficult response type, a mismatching action, whenever the SR pairing is imbalanced. This prior bias, which participants then override if needed, could be evident in the very earliest initiation of a response—if the configuration of the gesture began by tending toward the opposing action regardless of the trial cue. This initial action may be subtle and then quickly corrected to the cued action. We did not acquire sufficiently detailed movement recordings to address this. Future experiments using motion capture video to tease apart the detailed kinematics of participants’ initial responses would illuminate this.

Inferential analyses indicated the reaction-time cost typically associated with SR incongruence was present only if congruence was unpredictable—where p(SRmatch) = 0.5. This concurs with previous research using SRC paradigms where the likelihood of congruence is held constant throughout the task—50:50 ratio. Reaction-time costs were otherwise reversed when the SR congruence likelihood was above (0.7 or 0.9) or below (0.1 or 0.3) chance, favouring quicker responses on incongruent trials. This result was only partly aligned to our hypotheses for reaction-time effects: we expected inhibition of mirrored responses (hence faster responses for incongruent trials) if this incongruence was expected, p(SRmatch) = 0.1 and 0.3. However, the faster RT results for incongruent responses when congruent SR pairs were predictable and more expected violated our expectations. Recent work by Gordon et al. (2020) compared blocks of mostly congruent trials (25% incongruent) against a mostly incongruent block (75% incongruent) to find the classic interference effect was attenuated when incongruent SR pairs were expected more than congruent. Their study focused on potential group differences between neurotypical and Autistic individuals yet found both groups displayed similar attenuation of interference effects when a block had mostly incongruent trials; and showed a maintenance of the classic SRC effect in their mostly congruent block. Our result partially accords with this finding in that we also report an attenuation of mirroring for predictable incongruence. However, contrary to the results from Gordon et al., for contexts where congruence was moderately or very likely, p(SR-match) = 0.7 or 0.9, the reaction-time mismatch costs was reversed. The classic interference effect was only present in the unpredictable context.

The restriction of the classic SRC effect to the unpredictable blocks in our experiment highlights the need to account for expectation and predictability in models of human mirroring. This effect is reflected on the trial- and block-dependent changes in volatility and learning rate inferred from our RT data by inverting the HGF model. In contrast, the classic RW model assumes that the cue–stimulus relationship is stable, and that a constant learning rate will be sufficient to weight the comparison of expectations to outcome necessary to learn the pattern of events. The superior performance of the HGF shows that in a volatile setting, human agents build an expectation about the relative stability of events when planning motor responses to observed actions and adjust their rate of learning accordingly. Put more formally, modelling these behavioural data with the HGF showed that priming of a congruent response occurred only when the environment was estimated to be more volatile. In contexts of lower volatility estimates, a priming of *counter-imitation* appeared to prevail. Our computational modelling indicates an advantage for dynamic learning, including estimates of the volatility of the environment, for capturing the mismatch behaviour in a changeable context. An ambition for future studies will be to improve the integration of the perceptual and response components of the model. This may yield additional outcomes, such as capturing the mismatch-cost reversal across different probability contexts.

Our paradigm is not a pure measure of automatic imitation, as it does not separate *spatial* SRC from *imitative* compatibility. Previous research suggests that imitative compatibility cannot be reduced to spatial compatibility effects (Boyer et al., 2012; Heyes, 2011), yet the two are related and can co-occur. Using the current paradigm, we cannot rule out that the observed changes to SRC effects across probability contexts were driven by spatial rather than imitative aspects of the SR pairing. The classic spatial compatibility effect is in the same direction as imitative compatibility— (i.e., an SR mismatch, whether spatial or imitative, will slow reaction times compared with a matched SR pair; Brass et al., 2000). The magnitude of spatial compatibility effects is often greater than that of imitative compatibility effects, and therefore spatial compatibility could be influencing reaction times. While spatial compatibility effects have been shown to be more persistent, imitative biases are more readily inhibited by top-down strategies (Cooper et al., 2013), which suggests that the current effects might be due to probabilistic contexts providing strategic means to control imitative, more than spatial influences of the SRC. Nonetheless, further investigation with a task integrating spatial and imitative cues would be required to disentangle their influences. Further studies of SR compatibility within probabilistic contexts would allow testing the extent to which predictive coding models may explain the distinct timing of spatial versus imitative SRC effects, given previous findings that spatial and imitative compatibility follow different time courses (Catmur & Heyes, 2011).

Real-world events are inherently unstable, and learning requires flexibility. Our probabilistic paradigm expanded on the bulk of automatic imitation and mirroring research which has relied on randomized (unpredictable) SR congruence. The HGF here incorporated three sources of uncertainty (Fig. 3) hierarchically, but the key strength is that the learning-rate at the second level (Fig. 3, orange panel) is continually updated according to estimates of environmental uncertainty (from the third level, Fig. 3, green panel). This dynamic, uncertainty-weighted updating in the HGF offers a distinct advantage over models which assume a fixed “ideal” learning rate because it incorporates the inherently unstable nature of the environment (Behrens et al., 2007). Further research will be needed to determine if our finding is generalizable to other instances of sensorimotor integration and other classic SR compatibility effects (Prinz, 1997; Posner, 1980).

Taking a systems-level view of the causal role played by the mirror neuron system in imitation (Heyes & Catmur, 2022), we positioned our paradigm as an instantiation of predictive coding within action mirroring. To interrogate this further, future research could employ a combination of functional neuroimaging and computational approaches (as per Auksztulewicz et al., 2017; Vossel et al., 2014; Vossel et al., 2015; Iglesias et al, 2013). Neural activity related to our imitation/counter-imitation task, combined with the subject-specific HGF parameters as regressors, would allow for an analysis of effective connectivity between regions of the mirror neuron system, action observation and higher-order executive control brain regions (Campbell et al., 2021). Such an extension on the current study could test the hypothesis that dampening of activity within ‘mirror’ regions depends on expectations and uncertainty about the likelihood of SR congruence. This approach would align with a recent review of mirror neuron research: “it turns out that mirror neurons contribute to complex control systems rather than dominating such systems or acting alone” (Heyes & Catmur, 2022, p. 163).

## Supplementary Information

ESM 1 (PDF 165 kb)
