# Supplementary material for: Reaction Time “Mismatch Costs” Change with the Likelihood of Stimulus–Response Compatibility
Source: Psychon Bull Rev. 2022 Aug 25;30(1):184–99. doi: 10.3758/s13423-022-02161-6 (PMC9971163; doi:10.3758/s13423-022-02161-6)
Supplement: Supplementary file 1 — (PDF 165 kb) [file 13423_2022_2161_MOESM1_ESM.pdf]

Supplementary Materials

"Automatic imitation effects are restricted to unpredictable contexts"
Campbell, Sherwell, Cunnington, Brown & Breakspear

S1. Missing data
Missing data was defined by failures to respond within the response period (i.e. within the duration of 1s movement stimulus). Either responding too early (anticipating the movie) or not responding within 1s resulted in a missing data point.

For the 30 participants who completed 10 blocks of the task, the mean percentage of missing trials by condition are presented below in Table S1a, with Table S1b showing this for the analysis sample of 28 participants. Note a third participant wasn't included in analyses because experimenter error lead to the task only being run for 6 not 10 blocks.

Table S1a: *Mean percentage missing trials for n=30 participants who completed the task.*

	Probability of SR Match (context)				
	0.1	0.3	0.5	0.7	0.9
Mismatch	5%	5%	5%	7%	4%
Match	5%	6%	5%	6%	5%

Table S1b: *Mean percentage missing trials for n=28 participants after excluding 2 participants 20% or more missing trials.*

	Probability of SR Match (context)				
	0.1	0.3	0.5	0.7	0.9
Mismatch	3%	2%	2%	3%	2%
Match	3%	4%	3%	2%	2%

Table S2: *Trial counts by conditions: SRC x probability of match*

SRC	Probability of SR Match (context)				
	0.1	0.3	0.5	0.7	0.9
Mismatch	72	56	40	24	8
Match	8	24	40	56	72

S2. Response Model estimated reaction times

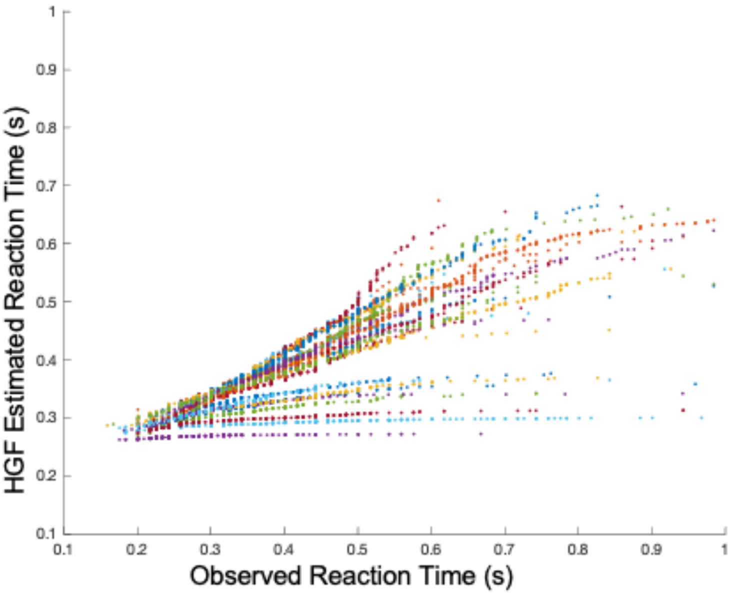

Figure S1. Observed versus fitted reaction times using the drift-diffusion model with parameters derived from the HGF perceptual model. Mean RTs are captured in all participants. However, in a subset of (8) participants, the longer tails (slower RTs) of the observed responses are under-estimated. The correlation coefficient for observed and estimated values is: R=0.8402 (95% CI: 0.8346, 0.8457).
